# Modulation of gut microbiota and immune response by soy peptides mitigates irinotecan induced intestinal toxicity

**DOI:** 10.3389/fphys.2025.1538733

**Published:** 2025-06-03

**Authors:** Yongfa Jing, Dongli Yan

**Affiliations:** ^1^ Department of Handan First Hospital, Handan, China; ^2^ Department of Hebei Runkang Medical Technology, Handan, China

**Keywords:** irinotecan, soy peptides, diarrhea, intestinal damage, gut microbiota, immune modulation

## Abstract

**Introduction:**

Irinotecan (CPT-11), a cornerstone chemotherapeutic agent for colorectal and pancreatic cancers, is limited by severe gastrointestinal toxicities, particularly diarrhea, which compromises treatment adherence and patient quality of life. Soy peptides (SPs), bioactive compounds with anti-inflammatory and prebiotic properties, have shown potential in enhancing intestinal barrier function. This study investigates SPs’ protective effects against irinotecan-induced intestinal injury, focusing on microbiota modulation, immune regulation, and mucosal repair.

**Methods:**

Female C57BL/6 mice were randomly divided into four groups (n = 10/group): Control, Irinotecan, Pre-SPs+ Irinotecan, and SPs+ Irinotecan. Diarrhea severity and body weight changes were monitored daily. Small intestinal injury was evaluated by hematoxylin and eosin (H&E) staining with epithelial damage scoring, while intestinal barrier integrity was assessed via Western blotting (WB) and immunohistochemistry. Serum levels of inflammatory cytokines (TNF-α and IL-6) were quantified using ELISA, and neutrophil infiltration was measured by flow cytometry. Fecal samples were subjected to 16S rRNA sequencing to analyze gut microbiota composition.

**Results:**

SPs intervention significantly reduced the incidence of diarrhea (*P* < 0.05) and attenuated body weight loss (*P* < 0.05) in mice. Histological analysis demonstrated that SPs restored intestinal architecture, as evidenced by reduced epithelial damage scores (*P* < 0.05), increased expression of tight junction proteins (occludin and ZO-1), and improved intestinal permeability (*P* < 0.05). Gut microbiota profiling revealed that irinotecan-induced dysbiosis was characterized by decreased α-diversity and enrichment of pathogenic taxa. SPs treatment restored microbial diversity and significantly elevated the abundance of beneficial genera, including *Lactobacillus* and *Bifidobacterium* (*P* < 0.05). Immunological assays further indicated that SPs suppressed pro-inflammatory cytokine levels (TNF-α and IL-6, *P* < 0.001) and reduced neutrophil infiltration (*P* < 0.05).

**Discussion:**

These findings suggest that soy peptides protect against irinotecan-induced intestinal toxicity through multiple mechanisms, including microbiota regulation, immune modulation, and intestinal barrier restoration, highlighting their potential as a therapeutic candidate for chemotherapy-induced intestinal damage.

## Introduction

Irinotecan (CPT-11), a broad-spectrum topoisomerase inhibitor approved in Japan in 1995 for cancer therapy, is widely used to treat metastatic or advanced solid tumors such as gastric, pancreatic, ovarian, and colorectal cancers (Yue et al., 2021). However, CPT-11 frequently induces gastrointestinal disturbances, including nausea, vomiting, and diarrhea, and may even lead to life-threatening symptoms like electrolyte imbalances, dehydration, and severe pain (Zhu et al., 2021; Wu et al., 2019). Diarrhea occurs in approximately 60%–80% of patients receiving CPT-11, with 22%–44% experiencing grade 3 or 4 severe diarrhea (de Man et al., 2018). These adverse effects can interrupt treatment and diminish patients’ quality of life and prognosis (Xiao et al., 2023).

CPT-11 exerts its therapeutic effects by its active metabolite, SN-38, which kills rapidly dividing cancer cells while also damaging normal blood cells, epithelial cells, and gut microbiota (Bailly, 2019). β-D-glucuronidases (GUSs) in gut bacteria can convert the relatively non-toxic glucuronide form of SN-38 back into its active form, SN-38, thereby enhancing the drug’s toxicity (Stringer et al., 2008; Forsgård et al., 2017). Additionally, CPT-11 induces apoptosis in jejunal and colonic crypt cells, leading to damage of the normal small intestinal mucosa (Gibson et al., 2003). The bacteria within the damaged mucosa can activate nuclear factor kappa-B (NF-κB) and the expression of pro-inflammatory cytokines, exacerbating tissue injury and resulting in symptoms such as diarrhea, bloating, and pain (Sonis et al., 2004; Keefe et al., 2004; Keefe, 2004). Therefore, the gut microbiota may play a critical role in the toxicity induced by CPT-11.

Peptides are polymers composed of various amino acid chains with a molecular weight of less than 10,000 Da. Soy protein can be partially hydrolyzed through enzymatic reactions to generate soy peptides (SPs) (Kim et al., 2021a). SPs not only provide nutritional benefits but also possess sensory functions such as taste, solubility, and emulsifying properties, in addition to exhibiting a range of physiological activities (Daher et al., 2020), including anticancer effects, immune enhancement, reduction of blood cholesterol and blood pressure, and improvement of calcium homeostasis (Messina and Messina, 2010; Rizzo and Baroni, 2018). Studies have shown that digestion of soy protein in the gastrointestinal tract produces bioactive peptides with antioxidant and anti-inflammatory properties. These SPs can induce apoptosis, downregulate oncogene expression, and enhance antiproliferative activity against cervical and breast cancer cells (Mora-Escobedo et al., 2009). Moreover, some research has found that SPs released by gastrointestinal proteases significantly inhibit the production of pro-inflammatory factors in macrophages induced by lipopolysaccharide (LPS), affecting cell viability (González-Montoya et al., 2018). These findings suggest that SPs may have potential preventive effects on colorectal cancer and inflammation, helping to maintain normal gastrointestinal physiological function.

This study systematically evaluates the impact of irinotecan treatment on gastrointestinal function and gut microbiota in mice through a combination of pathological analysis, Western blot (WB), flow cytometry, and high-throughput omics technologies. Additionally, the study investigates the potential therapeutic effects of soy peptide intervention in preventing and mitigating irinotecan-induced gastrointestinal toxicity. The findings are expected to provide a scientific basis and novel intervention strategies for managing the side effects of cancer chemotherapy.

## Materials and method

### Animals and experimental design

Forty six-week-old female C57BL/6 mice were purchased from GemPharmatech Co., Ltd. (SCXY(Su)2018-0008). The mice were housed in an individually ventilated cage system, with controlled conditions (temperature: 24°C ± 2°C, humidity: 50%–60%, 12-h light/12-h dark cycle). After a one-week acclimatization period with *ad libitum* access to food and water, the experiments were initiated. All procedures were conducted in compliance with the ethical guidelines of the Handan People’s Hospital Ethics Committee and approved by the committee (Approval No. 2023-K-040).

After a one-week acclimatization period, the 40 mice were randomly assigned to four groups: Control, Irinotecan (Irinotecan, I), Pre-intervention with Soy Peptides (Pre-SPs + Irinotecan), and Soy Peptide Intervention (SPs + Irinotecan). Mice in the I, Pre-SPs + Irinotecan, and SPs + Irinotecan groups received intraperitoneal injections of irinotecan (100 mg/kg, CAS:136572-09-3, Aladdin, China) once daily for 4 consecutive days to induce intestinal mucositis, while Control group mice received an equivalent volume of saline as a control. To assess the preventive effects of soy peptides on irinotecan-induced adverse reactions, the Pre-SPs + Irinotecan group began drinking soy peptide solution (provided by Hebei Runkang Medical Technology Co., Ltd.) 1 week prior to irinotecan treatment and continued until the end of the irinotecan injection period, with free access to drinking water. The SPs + Irinotecan group began soy peptide consumption on the day of irinotecan injection and continued until the experiment ended. Mice in the I and Control groups had normal access to drinking water.

During the experiment, the body weight and diarrhea status of the mice were recorded daily. The severity of diarrhea was scored based on the following criteria (Riera and Páez, 2021): 0 points for normal (normal stool or no diarrhea symptoms); 1 point for mild (slightly wet or soft stool); 2 points for moderate (wet, unformed stool with perianal staining); and 3 points for severe (watery stool with perianal staining). Using this scoring system, the researchers assessed the severity of diarrhea in the mice daily and recorded any changes.

### Intestinal histopathological analysis

All experimental procedures complied with animal welfare and ethical guidelines. At the study endpoint, mice were anesthetized with carbon dioxide until unresponsive, followed by cervical dislocation for euthanasia. After collection of small intestinal tissue, samples were washed and fixed with tissue fixative (G1101, Sevier, China) to preserve tissue integrity. Fixed samples were dehydrated, embedded in paraffin, and sectioned to a thickness of 4 µm. The sections were stained with hematoxylin and eosin (H&E), and morphological observations were conducted using an upright optical microscope (Nikon Eclipse Ci, Nikon, Japan) and imaging system (Nikon DS-U3, Nikon, Japan).

To evaluate the ratio of duodenal villi to crypts, villus height (from the tip of the villus to the villus-crypt junction) and crypt depth (the depth of invagination between adjacent villi) were measured using a microscope and image analysis software, ensuring data accuracy and reproducibility of the experimental results.

Mucosal damage was scored using a scale of 0–3: 0 points for normal mucosal structure; 1 point for mild focal or diffuse inflammation in the lamina propria with intact epithelial structure, mild edema, and congestion; 2 points for moderate focal or diffuse inflammation with epithelial disruption, moderate edema and congestion, primarily affecting the mucosa and villus tips; and 3 points for severe focal or diffuse inflammation, significant epithelial damage, severe edema, moderate congestion, with the formation of abscesses in the villi or crypts (if present).

Cell infiltration was scored as follows: 0 points for no cell infiltration; 1 point for localized infiltration limited to the mucosa; 2 points for moderate cell infiltration involving both the mucosa and submucosa; and 3 points for severe cell infiltration affecting the mucosa, submucosa, and muscularis layers.

Epithelial inflammation was scored as follows: 0 points for no signs of epithelial inflammation; 1 point for rare inflammation, with occasional leukocyte infiltration; 2 points for moderate inflammation, characterized by numerous leukocytes infiltrating the epithelium; and 3 points for severe inflammation, with a large number of leukocytes diffusely present in the epithelium.

### Western blot analysis

Total protein was extracted from mouse small intestine tissue using RIPE lysis buffer (P0013B, Beyotime, China) under ice-cold conditions. The extracted proteins were separated by SDS-PAGE and transferred to a polyvinylidene fluoride (PVDF) membrane (Merck Millipore, IPVH00010, United States) at 100 V for 90 min. After transfer, the PVDF membrane was incubated overnight at 4°C with primary antibodies against occludin (1:1,000, ab216327, Abcam, United Kingdom) and ZO-1 (1:1,000, ab307799, Abcam, United Kingdom) to ensure proper binding of the antigen to the antibody. Subsequently, the membrane was incubated with HRP-conjugated secondary antibody, goat anti-rabbit IgG H&L (HRP) (1:10,000, ab6721, Abcam, United Kingdom) at room temperature for 1 h for chemiluminescent detection. Protein bands were visualized using a gel imaging system (Thermo Scientific, iBright FL1000, United States), and band quantification was performed using ImageJ software, employing integrated density values and background signal correction to improve result reliability.

### Flow cytometry

After anesthetizing the mice, blood was collected into anticoagulant tubes and then treated with red blood cell lysis buffer to lyse the red blood cells. After centrifugation, the supernatant was discarded, and the cell pellet was retained for cell counting. Neutrophils were then isolated using Percoll gradient centrifugation, and the cell count in the resulting single-cell suspension was determined. Within 1 h of blood collection, cells were stained with 2 μg/mL of anti-mouse Ly6G-FITC antibody (clone 1A8, BD Pharmingen) for 15 min at room temperature in the dark. Following staining, NH4Cl lysis buffer was added, and the mixture was gently inverted and incubated for 30 min to lyse any remaining red blood cells. After staining, samples were analyzed by flow cytometry (AC6, BD Biosciences), with excitation at 488 nm and detection through the FITC channel (530/30 nm).

### Inflammatory factor analysis

This study used IL-6 (EK206, Multi Sciences, China) and TNF-α (EK282HS, Multi Sciences, China) ELISA kits to quantitatively measure the concentrations of IL-6 and TNF-α in mouse intestinal tissues. The experiment strictly followed the instructions provided by the ELISA kits to ensure the accuracy and reproducibility of the results. Specifically, the intestinal tissue from mice was first homogenized and centrifuged, and the supernatant was collected as the sample. Both the sample and standards were added to the ELISA plate wells for incubation with the capture antibody. The plate was then washed multiple times to remove non-specific binding. Next, a detection antibody was added, and incubation continued. The color reaction was developed by adding the enzyme substrate solution, and the reaction was stopped. Absorbance was measured at the specified wavelength using a microplate reader. IL-6 and TNF-α concentrations in the samples were calculated based on the standard curve. All experiments were repeated at least three times to ensure data reliability and statistical significance.

### Liver function analysis

This study assessed mouse liver function by isolating mouse serum and using commercially available assay kits for aspartate aminotransferase (C010-2-1, Nanjing JianCheng, China), alanine aminotransferase (C009-2-1, Nanjing JianCheng, China), alkaline phosphatase (A059-2-2, Nanjing JianCheng, China), and total bilirubin (BC5185, Solarbio, China). The experiments were strictly conducted according to the instructions provided with the kits to ensure accuracy and reproducibility of the results. All assays were performed in triplicate to ensure data reliability and statistical significance.

### 16S rRNA gene sequence analysis

16S rRNA sequencing was performed as previously described. Briefly, gut bacterial DNA was extracted from the cecum contents of mice using the Qiagen DNA Stool Mini extraction kit (Qiagen GmbH, Hilden, Germany), following the manufacturer’s instructions. The V4 region of the 16S rRNA gene was amplified using the forward primer 515 F (5′-ACTCCTACGGGAGGCAGCA-3′) and reverse primer 806 R (5′-GGACTACHVGGGTWTCTAAT-3′). Samples were barcoded and pooled to construct the sequencing library. Paired-end 2 × 250 bp reads were generated using the Illumina MiSeq platform (Illumina, San Diego, CA), and merged using FLASH. Reads were assigned to individual samples based on their unique barcodes. High-quality clean tags were obtained by applying specific filtering conditions, and the sequences were clustered into Operational Taxonomic Units (OTUs) using USEARCH, with a 97% similarity threshold. The unique representative sequences of the OTUs were obtained and taxonomically classified using the Greengenes database via the Ribosomal Database Project (RDP) Classifier. Alpha diversity was assessed using Mothur, and beta diversity was analyzed using QIIME.

### Statistical analysis

Data are presented as mean ± standard deviation (SD). Group differences were assessed using one-way analysis of variance (ANOVA). If significant differences were found by ANOVA, *post hoc* comparisons were performed using Tukey’s multiple comparison test to determine specific group differences. All statistical analyses were conducted using GraphPad Prism version 10.2 (GraphPad Software, LLC, United States) and R version 3.4 (R Foundation for Statistical Computing, Austria). Statistical significance was set at p < 0.05.

## Result

### Soybean peptides alleviates irinotecan-induced diarrhea and weight loss

**FIGURE 1:**
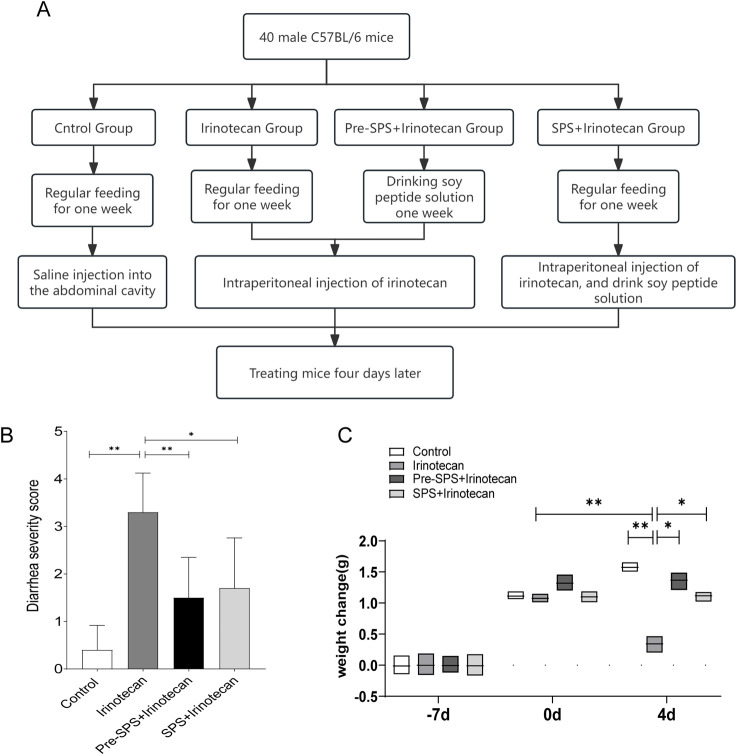
Soybean Peptides Alleviate Weight Loss and Diarrhea Induced by CPT-11 in Mice **(A)** The process of animal modeling. **(B)** Diarrhea Score in Mice. **(C)** Body Weight Change in Mice. Data represent mean ± standard deviation (SD). n = 10 per group. *P < 0.05, **P < 0.01.

This study aimed to evaluate the potential protective effect of soy peptides in alleviating irinotecan-induced adverse reactions. The results demonstrated that the irinotecan-treated mice had significantly higher diarrhea scores (Figure 1B), indicating substantial gastrointestinal toxicity induced by the drug, which exacerbated diarrhea. In contrast, the soy peptide-treated mice exhibited significantly lower diarrhea scores compared to those receiving irinotecan alone, suggesting that soy peptides may improve intestinal function and mitigate chemotherapy-induced gastrointestinal side effects, possibly through their anti-inflammatory effects.

Furthermore, irinotecan-treated mice showed a significant reduction in body weight (Figure 1C), with a markedly greater weight loss compared to the control group. This weight loss is likely associated with irinotecan-induced damage to the intestinal epithelium and resulting gastrointestinal dysfunction. Encouragingly, the soy peptide-treated mice experienced significantly less weight loss compared to the irinotecan group, indicating that soy peptides may help alleviate irinotecan-induced body weight changes by improving intestinal barrier function and reducing gastrointestinal inflammation.

### Protective effect of soybean peptides on irinotecan-induced intestinal mucosal injury

**FIGURE 2:**
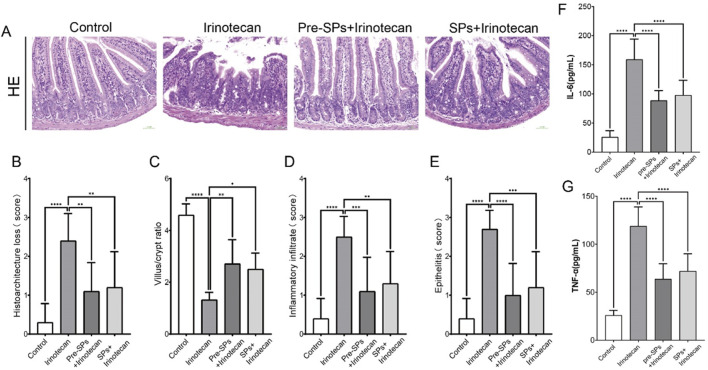
Soybean peptides protect mouse intestinal tissues from irinotecan-induced damage. **(A)** H&E staining of the ileal tissue in mice (30×). **(B)** Damage score of the ileal mucosal tissue. **(C)** Villus/crypt ratio. **(D)** Inflammatory infiltration. **(E)** Epithelial inflammation. **(F)** Expression of IL-6 in intestinal tissue. **(G)** Expression of TNF-α in intestinal tissue. Each group, n = 10. *P < 0.05, **P < 0.01, ***P < 0.001, ****P < 0.0001.

In this study, the researchers evaluated the protective effect of soy peptides on irinotecan-induced intestinal injury in mice through histopathological analysis of the distal ileum stained with hematoxylin and eosin (H&E). The results showed that in the control group, the intestinal mucosa exhibited a well-preserved structure, with typical villus morphology and tightly and regularly arranged epithelial cells (Figure 2A). However, irinotecan-treated mice displayed marked intestinal mucosal damage, including significant infiltration of inflammatory cells, loss of crypt structure, and disordered arrangement of epithelial cells, resulting in a significantly elevated histopathological score (Figure 2B).

In the soy peptide intervention group, these histopathological abnormalities in the intestinal tissue were significantly improved. The villus and crypt structures gradually recovered, and the histopathological score decreased notably, indicating that soy peptides provide a certain degree of protection against irinotecan-induced intestinal injury. Further analysis revealed that, compared to the control group, the villus-to-crypt ratio in the irinotecan-treated mice was significantly reduced, whereas following soy peptide intervention, the villus-to-crypt ratio was restored. This further supports the potential protective role of soy peptides in maintaining the integrity of the intestinal structure (Figures 2C–E).

Additionally, ELISA was used to measure the levels of pro-inflammatory cytokines IL-6 and TNF-α in the intestinal tissues of mice. The results showed that irinotecan treatment significantly increased the levels of IL-6 and TNF-α (Figures 2F,G), indicating that irinotecan induced a significant inflammatory response in the gut. The elevation of these pro-inflammatory cytokines may further exacerbate intestinal mucosal damage, impairing nutrient absorption and intestinal barrier function. Notably, soybean peptide intervention significantly reduced the expression levels of IL-6 and TNF-α (Figures 2F,G), suggesting that soybean peptides inhibit the release of inflammatory mediators and help alleviate the inflammation induced by irinotecan, thereby protecting gut function.

### Soybean peptides exert a protective effect by improving intestinal permeability

**FIGURE 3:**
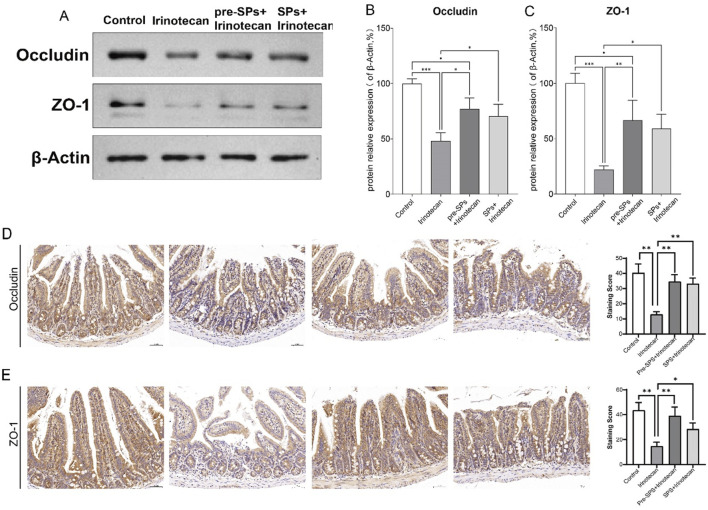
Soybean Peptides Maintain Intestinal Permeability in Mice. **(A)** Western blot analysis shows the expression of ZO-1 and Occludin in mouse ileum tissue. **(B,C)** ImageJ analysis indicates that the expression of ZO-1 and Occludin in the soybean peptide group was significantly higher than that in the irinotecan group, approaching the levels of the control group. Data are presented as mean ± SD, n ≥ 3 per group. **(D,E)** Representative immunohistochemical images of intestinal tissue show the expression of Occludin and ZO-1, with staining intensity quantified for evaluation (scale bar = 50 μm; magnification: ×25). *P < 0.05, **P < 0.01, ***P < 0.001.

In this study, the expression levels of Occludin and ZO-1 in mouse intestinal tissue were assessed via Western blot (WB). As shown in Figure 3, the expression of Occludin and ZO-1 was significantly reduced in the irinotecan-treated group, indicating a disruption of intestinal barrier function (Figures 3A–C). However, after intervention with soybean peptides, the expression of Occludin and ZO-1 was significantly increased, approaching the levels observed in the control group (Figures 3A–C).

Next, we assessed the effect of soy peptides on the expression of tight junction proteins Occludin and ZO-1 in intestinal tissues using immunohistochemistry. Compared to the control group, irinotecan treatment significantly reduced the expression of both Occludin and ZO-1 (Figures 3D,E). In contrast, supplementation with soy peptides markedly restored their expression levels (Figures 3D,E). This suggests that soybean peptides can partially restore the expression of tight junction proteins and thereby improve intestinal barrier function.

### Soybean peptides restore irinotecan-induced intestinal flora and dysbiosis

**FIGURE 4:**
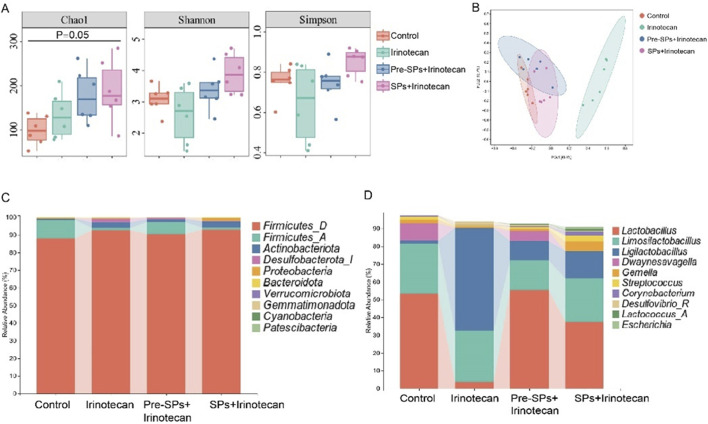
Soybean Peptides Restore Irinotecan-Induced Dysbiosis of the Gut Microbiota. **(A)** The α-diversity of the gut microbiota in the four groups of mice was evaluated using the Chao1, Shannon, and Simpson indices. **(B)** Principal coordinate analysis (PCoA), based on the Bray-Curtis distance matrix, shows differences in the gut microbiota structure among the different treatment groups. **(C)** The bar chart displays the differences in the gut microbiota composition at the phylum level. **(D)** The bar chart shows the differences in the gut microbiota composition at the genus level.

This study utilized 16S rDNA sequencing to conduct a comprehensive analysis of the changes in the intestinal microbiota of mice following irinotecan treatment and to investigate the potential protective effects of soybean peptides on the gut microbiome. The α-diversity of the intestinal microbiota was assessed using Chao1, Shannon, and Simpson indices. The Chao1 index reflects microbiota richness, while the Shannon and Simpson indices provide insight into microbiota diversity. The results revealed that irinotecan treatment significantly changed the Chao1, Shannon, and Simpson indices, indicating a marked variation in α-diversity (Figure 4A). In contrast, soybean peptide intervention notably improved these indices, suggesting that soybean peptides play a key role in restoring both the richness and diversity of the microbiota. This improvement effectively mitigates the disruption of the gut microbiome induced by irinotecan, highlighting its potential as a protective agent for the intestinal barrier.

Further β-diversity analysis, employing principal coordinate analysis (PCoA) based on the Bray-Curtis distance matrix, revealed significant shifts in the structure of the gut microbiota. The PCoA plot (Figure 4B) demonstrated a distinct separation between the Control and Irinotecan groups, indicating that irinotecan treatment profoundly altered the gut microbiota structure. The microbiota composition in the soybean peptide intervention group was notably different from that of the Irinotecan group, suggesting that the regulatory effects of soybean peptides contribute to the restoration of gut microbiota balance and, to some extent, reverse the dysbiosis induced by irinotecan treatment.

Based on the analysis of the gut microbiota bar charts and LefSe results, the study further investigated the impact of soybean peptides on specific microbial populations. The bar chart (Figure 4C) revealed that irinotecan treatment significantly disrupted the structure of the gut microbiota, particularly affecting the relative abundance of Firmicutes and certain beneficial genera. Soybean peptide intervention partially restored the abundance of these microbial populations. The restoration of Firmicutes is of particular physiological significance, as this phylum is widely recognized to be closely associated with host energy metabolism and intestinal barrier function.

**FIGURE 5:**
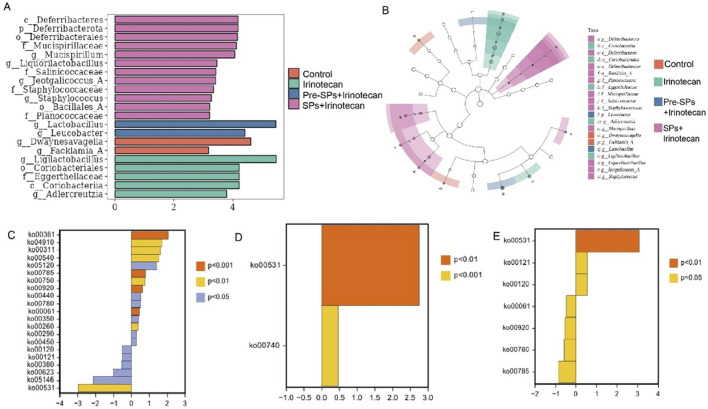
Differential Microbial Taxa and Metabolic Pathways Between Groups. **(A,B)** Differential bacterial taxa identified by LEfSe analysis: **(A)** LDA bar chart (LDA ≥3) showing significantly different bacterial taxa. **(B)** Taxonomic cladogram, where the node size corresponds to the average relative abundance of taxonomic units. Hollow nodes indicate no significant differences between groups, while colored nodes represent significant differences, with higher abundance in the respective group. **(C–E)** Differential metabolic pathway analysis between groups: **(C)** Differential metabolic pathways between the Control and Irinotecan groups. **(D)** Differential metabolic pathways between the Irinotecan and Pre-SPs + Irinotecan groups. **(E)** Differential metabolic pathways between the Irinotecan and SPs + Irinotecan groups. Note: Orange (*P* < 0.001), Yellow (*P* < 0.01), Blue (*P* < 0.05). Positive values on the x-axis indicate upregulation relative to the control group, while negative values indicate downregulation.

At the genus level (Figure 4D), irinotecan treatment significantly reduced the abundance of beneficial genera such as *Lactobacillus*, *Dwaynesavagella*, and *Facklamia*, while increasing the abundance of potentially pathogenic genera like *Ligilactobacillus*, *Desulfovibrio*, and *Paramuribaculum*. LefSe analysis showed similar results, with beneficial groups such as *Lactobacillus* enriched in the Pre-SPs + Irinotecan group and *Ligilactobacillus* enriched in the Irinotecan group (Figures 5A,B). The observed alterations indicate that irinotecan administration potentially disrupts microbial homeostasis, while soy peptides demonstrate the capacity to reestablish equilibrium in the gut microbiota. This finding underscores the protective function of soy peptides in modulating gut microbiota composition and promoting intestinal health.

Functional prediction based on the KEGG database was performed to investigate the metabolic potential differences of the gut microbiota among the different treatment groups using the metagenomeSeq method. A total of 21 metabolic pathways with significant differences were identified between the Control and Irinotecan groups (Figure 5C). Among these, 15 pathways, including *Chlorocyclohexane and chlorobenzene degradation*, were significantly upregulated in the Irinotecan group, while 6 pathways, including *Glycosaminoglycan degradation*, were downregulated. These differences suggest that irinotecan may alter the functional metabolic capacity of the gut microbiota by modulating specific metabolic pathways, thereby further affecting host health.

Soybean peptide intervention partially restored the changes in metabolic pathways induced by irinotecan. In the Pre-SPs + Irinotecan group, two key metabolic pathways, including *Glycosaminoglycan degradation*, were upregulated (Figure 5D). In the SPs + Irinotecan group, soybean peptides upregulated three metabolic pathways, including *Glycosaminoglycan degradation*, while downregulating pathways associated with fatty acid metabolism, such as *Fatty acid biosynthesis* (Figure 5E, Table 1). These results further suggest that soybean peptides not only regulate the composition of the gut microbiota but may also alleviate irinotecan-induced gut dysfunction by influencing its metabolic potential.

**TABLE 1:** Pathyway name.

Pathway	Name
ko00061	Fatty acid biosynthesis
ko00120	Primary bile acid biosynthesis
ko00121	Secondary bile acid biosynthesis
ko00260	Glycine, serine and threonine metabolism
ko00290	Valine, leucine and isoleucine biosynthesis
ko00311	Penicillin and cephalosporin biosynthesis
ko00350	Tyrosine metabolism
ko00361	Chlorocyclohexane and chlorobenzene degradation
ko00380	Tryptophan metabolism
ko00440	Phosphonate and phosphinate metabolism
ko00450	Selenocompound metabolism
ko00531	Glycosaminoglycan degradation
ko00540	Lipopolysaccharide biosynthesis
ko00623	Toluene degradation
ko00740	Riboflavin metabolism
ko00750	Vitamin B6 metabolism
ko00780	Biotin metabolism
ko00785	Lipoic acid metabolism
ko00920	Sulfur metabolism
ko04910	Insulin signaling pathway
ko05120	Epithelial cell signaling in *Helicobacter pylori*infection
ko05146	Amoebiasis

### Soybean peptides reduce intestinal inflammation

**FIGURE 6:**
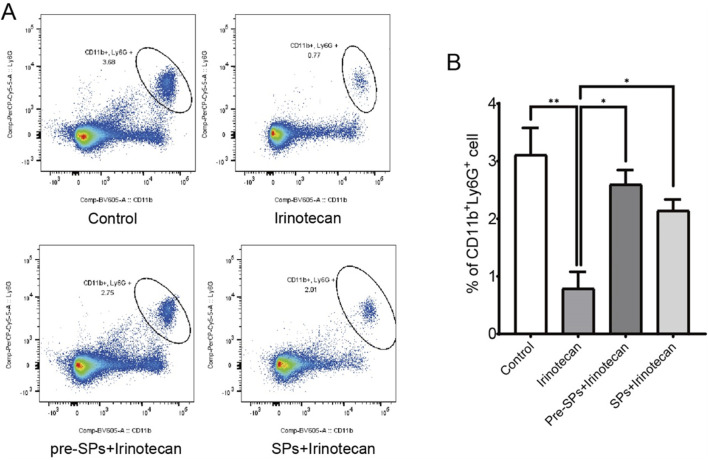
Soybean peptides reduce inflammation in mice. **(A,B)** Soybean peptides restore the reduction in neutrophil count induced by CPT-11 treatment. Note: *P < 0.05, **P < 0.01.

Flow cytometry was used to assess the number of neutrophils in the peripheral blood of mice. The results indicated that irinotecan treatment significantly reduced the number of neutrophils in the peripheral blood (Figures 6A,B). However, after soybean peptide intervention, the neutrophil count was restored to levels close to those of the control group. This suggests that soybean peptides may play a positive role in maintaining immune cell balance (Figures 6A,B).

### Soybean peptides alleviate irinotecan-induced liver damage

**FIGURE 7:**
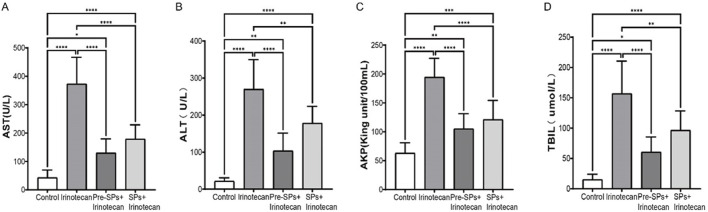
Soybean Peptides Alleviate Irinotecan (CPT-11)-Induced Hepatotoxicity. **(A)** Expression levels of AST in serum. **(B)** Expression levels of ALT in serum. **(C)** Expression levels of ALP in serum. **(D)** Expression levels of TBIL in serum. Note: Each group n = 10. *P < 0.05, **P < 0.01, ***P < 0.001, ****P < 0.0001.

To investigate the potential impact of soybean peptides on liver function, this study measured the levels of aspartate aminotransferase (AST), alanine aminotransferase (ALT), alkaline phosphatase (ALP), and total bilirubin (TBIL) in the serum of mice. The results indicated that irinotecan treatment significantly increased the levels of AST, ALT, ALP, and TBIL (Figures 7A–D), suggesting that irinotecan may cause liver damage. This elevation reflects hepatocellular injury and impaired liver function, particularly in terms of liver metabolism and detoxification.

Notably, after soybean peptide intervention, the levels of these biomarkers were significantly improved. Soybean peptides partially restored the levels of AST, ALT, ALP, and TBIL (Figures 7A–D), suggesting their potential role in protecting liver function. ALT and AST are sensitive markers of hepatocellular injury, while ALP and TBIL are associated with bile duct function and liver metabolism. The improvement of these biochemical markers by soybean peptides indicates that they may alleviate irinotecan-induced liver damage, possibly through their antioxidant and anti-inflammatory properties.

## Discussion

Irinotecan (CPT-11) is a broad-spectrum anticancer drug, but its clinical application is limited by severe side effects, including delayed-onset diarrhea, leukopenia, and nephrotoxicity (Riera and Páez, 2021). The onset of diarrhea is closely associated with impaired intestinal barrier function and changes in the gut microbiota (Lee, 2015; Landy et al., 2016). Soybean peptides, bioactive components extracted from soybeans, possess a variety of biological activities, including regulation of intestinal function, antioxidant effects, antithrombotic properties, immune enhancement, and promotion of liver function (Wen et al., 2022; Kim et al., 2021b; Kim et al., 2021c). Therefore, this study utilized a CPT-11-induced diarrhea mouse model to evaluate the potential of soybean peptides in alleviating CPT-11-induced intestinal and liver damage.

The results showed that CPT-11 treatment significantly caused a decrease in body weight and an increase in the severity of diarrhea, with the diarrhea score more than doubling, which is consistent with previous studies (Li et al., 2023). Treatment with soybean peptides effectively reversed the weight loss induced by CPT-11 (*p* < 0.05) and alleviated the symptoms of diarrhea. Notably, mice pretreated with soybean peptides exhibited less weight loss, suggesting that soybean peptides have significant potential in mitigating chemotherapy-related side effects. CPT-11 treatment induced marked pathological changes in the mouse intestine, including a reduction in the villus/crypt ratio, epithelial disruption, and cellular infiltration (Wang et al., 2019; Boeing et al., 2021). However, soybean peptide intervention significantly improved these pathological alterations.

In this study, we measured the levels of pro-inflammatory cytokines IL-6 and TNF-α in intestinal tissues and found that CPT-11 treatment markedly upregulated their expression. This aligns with prior reports implicating inflammation as a key contributor to CPT-11-induced intestinal toxicity (Wang et al., 2019; Boeing et al., 2021; Paterson et al., 2023). However, soybean peptide intervention significantly reduced the levels of these pro-inflammatory cytokines, indicating its active role in suppressing inflammation. Previous studies have demonstrated that active components from soy, such as isoflavones and peptides, possess significant anti-inflammatory effects, primarily by inhibiting the activation of inflammatory pathways like NF-κB, which in turn reduces the release of inflammatory factors and alleviates tissue damage (Karas et al., 2022).

CPT-11 treatment significantly decreased the expression of Occludin and ZO-1 in the mouse intestine, increased intestinal permeability, and subsequently activated the intestinal inflammatory response (Arifa et al., 2016). However, soybean peptide intervention significantly restored the expression levels of Occludin and ZO-1, reduced intestinal permeability, and improved the intestinal barrier damage. Maintaining intestinal barrier function is crucial for intestinal homeostasis (Lee, 2015). Tight junctions (TJs) are central components of the intestinal barrier, maintaining the integrity of the intestinal structure through transmembrane proteins such as Occludin, Claudin-1, and ZO-1 (Buckley and Turner, 2018). This suggests that soybean peptides improve intestinal barrier function by repairing tight junction proteins.

The results of this study demonstrate that CPT-11 treatment significantly decreased intestinal microbiota diversity and reduced the abundance of beneficial bacteria such as *Firmicutes* and *Lactobacillus* (Meng et al., 2022), while increasing the abundance of *Actinobacteria* and potentially harmful bacteria like *Eggerthellaceae* and *Adlercreutzia* (Moreira et al., 2012). Soybean peptide intervention was able to restore the diversity and structure of the gut microbiota, increasing the abundance of symbiotic bacteria and decreasing the abundance of harmful bacteria, thereby improving the dysbiosis induced by CPT-11. It has been shown that the gut microbiota is an important component in maintaining intestinal homeostasis, regulating barrier integrity and metabolic processes (Wang et al., 2019; Alexander et al., 2017; Nicholson et al., 2005). The efficacy of soy peptide therapy may be attributed to the ability of soybean peptides to promote the colonization of beneficial bacteria while inhibiting the growth of harmful bacteria, thus indirectly alleviating intestinal inflammation and barrier dysfunction.

The results of this study indicate that CPT-11 treatment significantly increased the serum levels of AST, ALT, ALP, and TBIL, suggesting liver dysfunction. Irinotecan (CPT-11) not only exerts toxic effects on the intestine but also leads to significant liver damage, especially in combination therapy regimens (Huang et al., 2020). AST and ALT are sensitive biomarkers of liver cell injury, indicating disruption of liver cell membrane integrity, while elevated ALP and TBIL levels reflect biliary dysfunction, indicating the broad impact of CPT-11 on liver metabolism and detoxification (Xiong et al., 2017). Soybean peptide intervention significantly reduced these biochemical markers, suggesting a protective effect on liver function. Early studies have also shown that soybean extracts exhibit anti-inflammatory and antioxidant activities in other liver injury models (Paterson et al., 2023), which aligns with the protective role of soybean peptides in alleviating CPT-11-induced liver damage observed in this study.

Moreover, metabolic pathway analysis revealed that CPT-11 treatment led to the downregulation of the Glycosaminoglycan (GAG) degradation pathway, while soybean peptide intervention upregulated this pathway. GAGs play an essential role in maintaining intestinal and liver homeostasis, participating in cytokine signaling, stabilization, and storage (Muramatsu and Muramatsu, 2008). The downregulation of GAG degradation may limit cytokine signaling, affecting the regulation of the inflammatory response and the tissue repair ability (Okita et al., 2009). On the other hand, the upregulation of the GAG degradation pathway by soybean peptides may help promote the appropriate release of cytokines and the clearance of metabolic waste products, thereby alleviating CPT-11-induced intestinal and liver damage and improving overall health (Eder et al., 2009). This suggests that soybean peptides, by regulating GAG metabolism, maintain the dynamic balance of the extracellular matrix and further exert their anti-inflammatory and tissue-protective effects.

In conclusion, this study demonstrates that soybean peptides play a significant role in alleviating the intestinal and liver toxicity induced by CPT-11. Soybean peptides not only effectively reduce the diarrhea and weight loss caused by CPT-11, but also counteract CPT-11-induced toxicity through various mechanisms. These include restoring intestinal barrier structure, increasing the expression of tight junction proteins, regulating the gut microbiota composition, and lowering pro-inflammatory cytokine levels. Additionally, the protective effect of soybean peptides on liver function indicates their broad physiological impact across multiple organ systems. This provides important theoretical support and application prospects for soybean peptides as an adjunctive therapy to mitigate the adverse effects of chemotherapy drugs and enhance their therapeutic efficacy.

## Data Availability

The original contributions presented in the study are included in the article/supplementary material, further inquiries can be directed to the corresponding author.
